# Effectiveness of Vaccination in Preventing COVID-19: A Community Study Comparing Four Vaccines

**DOI:** 10.3390/vaccines11030544

**Published:** 2023-02-24

**Authors:** Zoran Kokić, Predrag Kon, Olgica Djurković-Djaković

**Affiliations:** 1 Community Health Centre Voždovac, 11010 Belgrade, Serbia; 2 Belgrade City Institute of Public Health, 11108 Belgrade, Serbia; 3 Institute for Medical Research, University of Belgrade, 11129 Belgrade, Serbia

**Keywords:** COVID-19, vaccination, vaccine effectiveness, BBIBP-CorV, BNT162b2, Gam-COVID-Vac, ChAdOx1

## Abstract

The course of the COVID-19 pandemic has been critically altered by the availability of vaccines. To assess the risk of COVID-19 in the vaccinated, as compared to the unvaccinated population, as well as the comparative effectiveness of the BBIBP-CorV (Sinopharm), BNT162b2 (Pfizer/BioNTech), Gam-COVID-Vac (Sputnik V) and ChAdOx1 (AstraZeneca) vaccines in the prevention of clinical infection, we carried out a retrospective study of the incidence of clinical COVID-19 in the Belgrade city municipality of Voždovac among both vaccinated and unvaccinated individuals during a 4-month period between 1 July and 31 October 2021. The study included all individuals with a symptomatic infection confirmed by a positive PCR and/or antigen test. Only those who received two vaccine doses were considered as vaccinated. The results showed that of the Voždovac population of 169,567, a total of 81,447 (48%) individuals were vaccinated by the end of the study. Vaccination coverage increased with age, ranging from 1.06% in those below age 18, to even 78.8% in those above 65 years of age. More than one half (57.5%) of all those vaccinated received BBIBP-CorV, while 25.2% received BNT162b2, 11.7% Gam-COVID-Vac and 5.6% ChAdOx1. The overall risk of infection of the vaccinated vs. the unvaccinated was 0.53 (95% CI 0.45–0.61). Compared to the incidence of COVID-19 of 8.05 per 1000 in the unvaccinated population, the relative risk in the vaccinated was 0.35 (95% CI 0.3–0.41). The overall VE was 65%, differing widely among age groups and by vaccine. VE was 79% for BNT162b2, 62% for BBIBP-CorV, 60% for ChAdOx1 and 54% for Gam-COVID-Vac. The VE for BBIBP-CorV and BNT162b2 increased with age. The obtained results demonstrate a significant overall effectiveness of anti-COVID-19 vaccination, which, however, varied significantly among the analyzed vaccines, and was the highest for BNT162b2.

## 1. Introduction

The coronavirus disease 2019 (COVID-19) has taken the world by storm since January 2020, followed by the World Health Organization (WHO) declaring it a pandemic in March 2020. More than 650 million cases, of which approximately 1% with fatal outcome, have been recorded to date [1]. Although hundreds of thousands of new cases are still reported daily at the global level, epidemiological measures have been relieved in most countries. This is due to numerous reasons, including social and economic ones, but the availability of vaccines was the critical factor that dramatically alleviated the global disease burden, allowing for the restoration of a close-to-normal life. The year 2021 was marked by mass vaccination throughout the world; with over 13 billion doses administered so far [1] across 184 countries [2] and with 162 doses given for every 100 people around the world by October 2022 [3], vaccination against COVID-19 is by far the largest vaccination campaign in history. As of December 2022, 13 vaccines have been granted emergency approval [4].

The vaccination campaign naturally instigated intensive research of its effectiveness in various populations across the world, both overall and for specific vaccines. A few months into the vaccination campaign, it became clear that vaccination was effective in terms of prevention of death, as well as of serious disease and hospitalization, even with the ongoing mutations of the virus. Indeed, several variants of concern (VoC) occurred and passed in the meantime, up to the current global spread of the Omicron variant and its many additional mutations [5]. Numerous studies have been and are being carried out around the world, a summary of which may be found at the International Vaccine Access Center [6], showing varying effectiveness of the different vaccines in different clinical settings, age groups, professional groups (healthcare workers), as well as according to the number of doses administered (two or more) and time after vaccination, as well as time of study in the context of the predominant viral variants [7,8,9,10,11,12,13,14]. However, to inform public health officials on epidemiological measures and policies, as well as to guide further efforts on the vaccine development and alterations, data from various settings and geographies are still needed for a full picture.

In Serbia, the vaccination campaign started in January 2021, and while at first only the population above 60 years of age was invited to get vaccinated, a few weeks later vaccines became available to adults in all age groups. At the start of the vaccination campaign, the vaccine available to the general population was BBIBP-CorV (Sinopharm, inactivated whole virus vaccine), while only weeks later individuals were able to choose from among this one and BNT162b2 (Pfizer-BioNTech, mRNA), and two vector vaccines, Gam-COVID-Vac (Sputnik V, Gamaleya) and ChAdOx1 (AstraZeneca). In May 2021, vaccination was broadened to encompass minors above age 12. Despite this early start and mass availability, the vaccination rate has not been as good as desired, with 47.7% of the population being fully vaccinated (and 27% receiving an additional dose) by December 2022.

We conducted a study to assess the risk of infection and overall vaccination effectiveness in vaccinated vs. unvaccinated individuals in the Belgrade city municipality of Voždovac during a period of five to nine months after the start of the vaccination. Furthermore, we assessed the comparative effectiveness of the four vaccines available at the time of the study, including BBIBP-CorV, BNT162b2, Gam-COVID-Vac and ChAdOx1 in terms of prevention of clinical infection.

## 2. Materials and Methods

### 2.1. Study Design, Setting and Study Population

A retrospective study of the incidence of severe acute respiratory syndrome coronavirus 2 (SARS-CoV-2) infection and COVID-19 in both vaccinated and unvaccinated individuals in the Belgrade city municipality of Voždovac was conducted during a 4-month period between 1 July and 31 October 2021. Data on the Voždovac population were obtained from the Statistical Office of the Republic of Serbia, while data on the vaccination status (total number of vaccinated individuals, by type of vaccine and number of doses received) were obtained from the National Registry for immunization against COVID-19 at the Institute for Public Health of Serbia. Data on the individuals affected by COVID-19 were collected by reviewing the medical records (database) of the COVID-19 unit at the local community health center (CHC Voždovac); this part of the study included all individuals with laboratory- (PCR or antigen test) confirmed symptomatic infection. For the analysis by vaccine type, individuals whose COVID-19 diagnosis was confirmed by a positive PCR test were interviewed by telephone by a trained staff epidemiologist (ZK) on their vaccination status. Those fully vaccinated (with two vaccine doses) were included in the study while individuals who had received only one vaccine dose at the time of the study were not considered vaccinated. The study was conducted at the time of the high transmission of the SARS-CoV-2 Delta variant both globally and locally (in Serbia), although the virus had not been typed in the patients in our sample.

The study followed the principles of the Declaration of Helsinki. Ethical approval was not required given the nature of the study, but the study has been reviewed by a local (CHC Voždovac) review body.

### 2.2. Detection of SARS-CoV-2

All individuals presenting at the CHC COVID-19 unit with symptomatic infection were tested for SARS-CoV-2. Nasopharyngeal and buccal swabs were collected at the COVID-19 Unit, and an antigen test (Panbio™ COVID-19 Ag Rapid Test, Abbott, Jena, Germany) was performed on site; if positive, it was considered confirmatory for SARS-CoV-2 infection. In the case of a negative antigen test, the swabs were expedited to the official regional PCR laboratory at the Institute of Virology, Vaccines and Sera “Torlak”, Belgrade for PCR testing, and the PCR result was considered diagnostic.

### 2.3. Statistical Analysis

All analyses were performed using an electronic database organized in the SPSS (version 11.5) statistical package (SPSS Inc., Chicago, IL, USA). The overall and vaccine-specific risks of infection were calculated by dividing the rates among vaccinated persons by the rates among unvaccinated persons; the results are presented as odds ratios (OR) with 95% confidence intervals (95% CI). Vaccine effectiveness (VE) was considered as effectiveness against symptomatic disease and was calculated according to the formula 1-V/N, where V is the disease incidence rate in vaccinated patients and N is the disease incidence rate in non-vaccinated individuals. VE was examined both overall and separately for each vaccine vs. the unvaccinated population, and for each vaccine in different age groups. A power analysis for comparison of the proportions showed a very high power of the study for the analysis of the incidence of COVID-19 in the vaccinated vs. the unvaccinated population (above 0.99), as well as for the analysis of VE by vaccine type (above 0.98).

## 3. Results

**Figure 1:**
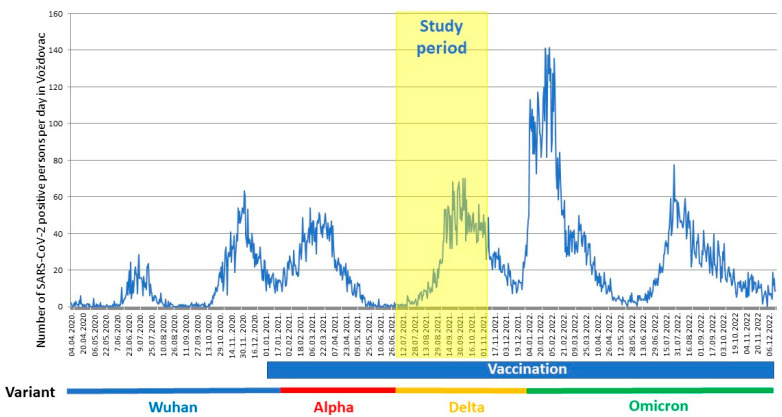
Timeline of the pandemic in the Voždovac municipality. Time of study (1 July–31 October 2021) depicted along with the vaccination timeline and dominant SARS-CoV-2 variant. The number of SARS-CoV-2 positive persons per day in Voždovac (from April 2020 through December 2022) as reported by the Institute of Public Health of Serbia (https://covid19.data.gov.rs, accessed on 27 December 2022).

Figure 1 shows the incidence of COVID-19 cases and the timeline of vaccination vs. the pandemic waves in the Belgrade city municipality of Voždovac.

The total population of Voždovac at the time of the study was estimated at 169,567. Of these, by 31 October 2021 a total of 81,447 individuals have been fully vaccinated, almost an exact half (48%). However, there was significant variation in the vaccination coverage among age groups, ranging from negligeable in minors (1.06%), to 46% in the 18–49 group and 63.9% in the 50–64 group, to even 78.8% in those older than 65. A majority of all those vaccinated received the BBIBP-CorV vaccine (57.5%), while 25.2% received the BNT162b2 vaccine, 11.7% the Gam-COVID-Vac (10.7%) and 5.6% the ChAdOx1 vaccine (Table 1). Importantly, none of the individuals vaccinated with any of the vaccines exhibited side effects other than immediate and transient reactogenicity, and no adverse reactions that would require medical assistance were reported to the relevant national authority (Medicines and Medical Devices Agency of Serbia).

During the study period, a total of 42,368 patients presented at the COVID-19 unit for consultation. A total number of 14,737 swabs was collected, of which 5396 (36.6%) tested positive (4656 positive antigen tests and 740 positive PCR results). Table 2 shows the incidence of COVID-19 cases in the vaccinated population, as compared to unvaccinated individuals. The overall risk of infection in the vaccinated vs. the unvaccinated population was 0.53 (95% CI 0.45–0.61), while the overall VE was 47% (95% CI 38.2–54.8). Analyzed according to age group, the relative risk decreased from 0.52 in the 18–49 group, to 0.43 in the 50–64 group and 0.27 in those above 65, resulting in a VE of 48%, 57% and 72% in the youngest, middle and oldest age groups, respectively (the group of under age 18 was not taken into account for these analyses due to the small numbers).

**Figure 2:**
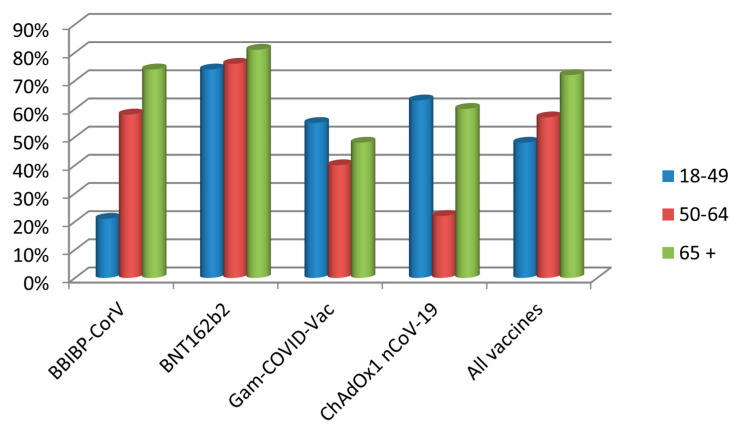
Vaccine effectiveness of the four vaccine types administered to the Voždovac community according to age group.

We next looked at the effectiveness of the individual vaccines (Table 3, Figure 2). Compared with an incidence of 8.05 per 1000 among the unvaccinated, the relative risk (RR) of infection in vaccinated individuals was 0.35 (95% CI 0.3–0.41). Accordingly, the overall VE was 65% (95% CI 58.8–69.9), but varying widely among individual vaccines, from 54% for Gam-COVID-Vac, 60% for ChAdOx1 and 62% for BBIBP-CorV, to even 79% for BNT162b2.

Importantly, the VE depended heavily on the age group (Table 4). For instance, although the overall effectiveness of BBIBP-CorV was 62%, it was only 21% effective in the 18–49 age group, but effectiveness increased to 58% in the 50–64 group and to 74% in the oldest age group. In contrast, the effectiveness of BNT162b2 varied only between 74% and 81%, but importantly, with both vaccines, the effectiveness increased with the age of the vaccinees. The two vector vaccines did not show such a pattern, in that the effectiveness of Gam-COVID-Vac had a decreasing trend with age, while ChAdOx1 showed a paradoxical poorest effectiveness in the middle age group, however within very broad CIs.

## 4. Discussion

The Belgrade city municipality of Voždovac provided an interesting setting for a population-based study for the occurrence of symptomatic COVID-19 as an almost exact half (48%) of its residents had been vaccinated at the time of the study. The results showed that the risk of infection was reduced by almost one half in the vaccinated vs. non-vaccinated individuals, and accordingly, the overall VE was 47%. Interestingly, however, VE increased with age, from 48% in the 18–49 years of age group to even 72% in the group older than 65. Compared with the incidence of COVID-19 of 8.05 per 1000 in the unvaccinated population, the RR in the vaccinated was 0.35. The overall estimated VE was 65%, but it differed widely according to vaccine and also among age groups. The VE ranged from 79% for BNT162b2, 62% for BBIBP-CorV and 60% for ChAdOx1, to 54% for Gam-COVID-Vac. Notably, the VE for BBIBP-CorV and BNT162b2 increased with age.

As numerous factors mentioned in the Introduction influence VE, it is hard to directly compare the VE results among different studies. Israeli studies that examined the early VE of BNT162b2 against PCR-confirmed infection showed it to be 94.5% and 93%, respectively [15,16]. Meta-analyses confirmed a VE of 95% for BNT162b2 in the pre-Delta period, but which decreased to 81% for the Delta variant [17,18]. A Hungarian nation-wide study that looked at the early VE (during the first five months of the vaccination campaign) of a similar set of vaccines as in our study, showed a VE against SARS-CoV-2 infection of 83% for BNT162b2, 86% for Gam-COVID-Vac, 71.5% for ChAdOx1 and 69% for BBIBP-CorV [19]. The lower VE estimates obtained in our study for all vaccines may be attributed to the predominant Delta variant, as well as to the longer time elapsed since vaccination (five to nine months since the beginning of vaccination). Indeed, lower VE against the Delta variant compared to the Alpha [20,21] variant, and the waning of VE over time have both been widely reported. Waning effectiveness of protection against SARS-CoV-2 infection was shown in another Hungarian study in the same nation-wide setting, especially six months after primary immunization [22], as well as in a similar nation-wide study from Greece [23]; this study also reported very high VE (>90%) in all age groups for BNT162b2 and ChAdOx1 against severe disease (requiring intubation) and death, but it did not examine VE against laboratory-confirmed COVID-19 cases. The data on the effectiveness of BBiBP-CorV are less abundant. Two Moroccan studies that looked at the effectiveness of BBIBP-CorV, again against SARS-CoV-2 severe disease/hospitalization, showed an early VE (within the first five months after the beginning of vaccination) against hospitalization of 90% [12]; and a VE ranging from 87–88% in the first three months, to 61% and 64% in the fifth and sixth month [24], which is quite similar to our finding at a comparable time after vaccination.

An interesting point of our study are the results obtained in older adults. First, it is of note that the vaccination rates increased with age, peaking at almost 80% in individuals over age 65; this obviously reflects the fact that older adults were the first to be invited for vaccination, but may also be on account of their awareness of being at highest risk of severe infection. Similarly, vaccination of above a half of the study group with BBIBP-CorV is due at least in part to its earlier availability, although it may also be attributed to a higher level of trust in older types of vaccines as compared to vaccines based on the mRNA platform; the latter were associated, particularly in the beginning, with a level of uncertainty driven by negative publicity in the media and on social media.

At the beginning of the pandemic, older adults were associated with a higher risk of SARS-CoV-2 infection and severe COVID-19, mostly due to co-morbidities as a major risk factor for poor prognosis [25]. Although vaccination critically changed the initially dire clinical outcome and case fatality rate of COVID-19-affected older adults, they remain the most vulnerable age group. In this respect, a most important finding of this study is a higher VE with age, particularly with the BNT162b2 and BBIBP-CorV vaccines. This is especially interesting since these two vaccines were received by a large majority (87%) of the examined population. The increase in the effectiveness of the BBIBP vaccine was particularly remarkable, as its effectiveness was only 21% in the 18–49 age group, but 58% in the 50–64 group and even 74% in the oldest age group. Although a reduced VE in the population aged above 60 was reported [24], the above-mentioned Hungarian study found the VE in individuals over 65 to be at least as good as in the younger population [22]. Since the ability to mount an adequate immune response following vaccination has been shown to be well preserved in older adults [26,27,28], increased VE in this population may be attributed to decreased exposure to the virus given that older categories of the population are the most inclined to closely follow the required epidemiological measures. Whatever the underlying reason(s), this is just as well since the highest proportion of the population in Serbia had received this vaccine, including in our study group.

However, the results we obtained in older adults may be compared to a similar population-based study carried out in adults above 60 years of age in the Vojvodina province of Serbia (just north of Belgrade), which showed higher VE for all the vaccines (87% for BBIBP-CorV, 95% for Gam-COVID-Vac and 99% for BNT162b2, and 89%) after the first dose of ChAdOx1 [29]. As the Petrović, et al. study was carried out in the first four months of the vaccination campaign, and the present study took place in a period of five to nine months after the beginning of the vaccination campaign, it may be considered to extend their data over the next few months; the lower VE in the present study demonstrates waning of the VE over time, but also the appearance of the SARS-CoV-2 Delta variant in circulation.

Our study’s major strength is its large scale as well as the comparison of four different vaccine types. However, there are several limitations to our study. One is the unknown precise time between vaccination and breakthrough infection, since the interviews took place within a four-month period (and the exact date of vaccination had not been recorded). Another limitation is the small number of PCR-confirmed infections in some of the age groups stratified by vaccine, particularly in the groups receiving the vector vaccines, rendering a lower power of analysis and thus possibly skewing the data. In addition, we here analysed the data before the appearance and continued presence of the Omicron variant, and before administration of the third and fourth vaccine doses; these are the matter of ongoing studies.

In conclusion, this study showed that vaccines successfully lowered the risk of SARS-CoV-2 infection at the community level. The presented results demonstrate significant overall effectiveness of anti-COVID-19 vaccination. However, VE varied significantly among the analysed vaccines, and was the highest for BNT162b2.

## Figures and Tables

**Table 1:** Vaccination coverage in the Belgrade municipality of Voždovac (on 31 October 2021) by vaccine type and age.

Age	Population	Vaccinated Population	Population Vaccinated with BBIBP-CorV	% Vaccinated Population with BBIBP-CorV	Population Vaccinated with BNT162b2	% Vaccinated Population with BNT162b2	Population Vaccinated with Gam-COVID-Vac	% Vaccinated Population with Gam-COVID-Vac	Population Vaccinated with ChAdOx1	% Vaccinated Population with ChAdOx1 nCoV-19
All	169,567	81,447 (48%)	46,815 (27.6%)	57.5%	20,506 (12.1%)	25.2%	9526 (5.6%)	11.7%	4600 (2.7%)	5.65%
0–17	29,380	312 (1.1%)	2 (0.01%)	0.64%	309 (1.05%)	99%	1 (0.003%)	0.32%	0 (0%)	0%
18–49	75,050	34,518 (46%)	14,333 (19.1%)	41.5%	13,259 (17.7%)	38.4%	3940 (5.25%)	11.4%	2986 (4%)	8.65%
50–64	31,677	20,256 (63.9%)	11,387 (35.9%)	56.2%	4562 (14.4%)	22.5%	3059 (9.65%)	15.1%	1248 (3.9%)	6.2%
≥65	33,460	26,361 (78.8%)	21,093 (7.1%)	80%	2376 (7.1%)	9%	2526 (7.65%)	9.6%	366 (1.1%)	1.4%

**Table 2:** Incidence of the PCR-confirmed COVID-19 in vaccinated and non-vaccinated individuals in the Belgrade municipality of Voždovac (on 31 October 2021).

Age	Number of Vaccinated	Number of COVID-19 Cases in the Vaccinated Population	Incidence of COVID-19 Cases in the Vaccinated Population (Per 1000 People)	Number of Unvaccinated	Number of COVID-19 Cases in the Unvaccinated Population	Incidence of COVID-19 Cases in the Unvaccinated Population (Per 1000 People)	Relative Risk (RR)	95% CI	Effectiveness	95% CI
All	81,447	231 (0.28%)	2.83	88,120	473 (0.54%)	5.37	0.53	0.45–0.61	47%	38.2–54.8
0–17	312	0	0	29,371	80 (0.27 %)	2.72	0			
18–49	34,518	111 (0.32%)	3.21	40,532	252 (0.62%)	6.21	0.52	0.414–0.646	48%	35.4–58.6
50–64	20,256	71 (0.35%)	3.5	11,421	93 (0.81%)	8.14	0.43	0.316–0.586	57%	41.4–68.4
≥65	26,361	49 (0.18%)	1.88	7099	48 (0.67%)	6.76	0.27	0.185–0.409	72%	59.1–81.5

**Table 3:** Effectiveness of the SARS-CoV-2 vaccines against symptomatic infection by vaccine type.

Vaccine	Population	Number of COVID-19 Cases	Incidence of COVID-19 Cases	Relative Risk (RR)	95% CI	Effectiveness	95% CI
Unvaccinated	58,740	473	8.05				
BBIBP-CorV	46,815	147	3.14	0.39	0.32–0.42	62%	53.1–67.6
BNT162b2	20,506	34	1.66	0.21	0.14–0.23	79%	70.9–85.5
Gam-COVID-Vac	9526	35	3.67	0.46	0.32–0.64	54%	35.7–67.6
ChAdOx1	4600	15	3.26	0.4	0.24–0.68	60%	32.4–75.8
All vaccines	81,447	231	2.83	0.35	0.3–0.41	65%	58.8–69.9

**Table 4:** Effectiveness of the SARS-CoV-2 vaccines against symptomatic infection by age.

Age	Number of Vaccinated	Number of COVID-19 Cases in the Vaccinated Population	Incidence of COVID-19 Cases in the Vaccinated Population (Per 1000 People)	Number of Non-Vaccinated	Number of COVID-19 Cases in the Unvaccinated Population	Incidence of COVID-19 Cases in the Unvaccinated Population (Per 1000 People)	Relative Risk (RR)	95% CI	Effectiveness	95% CI
**BBIBP-CorV**										
18–49	14,333	71	4.95	40,532	252	6.22	0.79	0.61–1.04	21%	−3.6–38.7
50–64	11,389	39	3.42	11,421	93	8.14	0.42	0.29–0.61	58%	38.9–71
≥65	21,093	37	3.75	7099	48	6.76	0.25	0.17–0.39	74%	60.2–83.1
**BNT162b2**										
18–49	13,259	22	1.65	40,532	252	6.22	0.26	0.17–0.41	74%	58.8–82.7
50–64	4562	9	1.97	11,421	93	8.14	0.24	0.12–0.48	76%	52–87.8
≥65	2376	3	1.26	7099	48	6.76	0.19	0.06–0.59	81%	40.1–94.2
**Gam-COVID-Vac**										
18–49	3940	11	2.79	40.532	252	6.22	0.45	0.25–0.82	55%	18–75.4
50–64	3059	15	4.9	11.421	93	8.14	0.6	0.35–1.04	40%	−3.7–65
≥65	2526	9	3.56	7.099	48	6.76	0.52	0.26–1.07	48%	−7.2–74.1
**ChAdOx1**										
18–49	2986	7	2.34	40,532	252	6.22	0.37	0.18–0.79	63%	20.2–82.2
50–64	1248	8	6.41	11,421	93	8.14	0.78	0.58–1.62	22%	−61.7–61.7
≥65	366	1	2.73	7099	48	6.76	0.4	0.06–2.92	60%	−191.9–94.4

## Data Availability

The data that support the findings of this study are available from the corresponding author upon reasonable request.
